# Romantic ideals, mate preferences, and anticipation of future difficulties in marital life: a comparative study of young adults in India and America

**DOI:** 10.3389/fpsyg.2014.01355

**Published:** 2014-12-02

**Authors:** Kathrine Bejanyan, Tara C. Marshall, Nelli Ferenczi

**Affiliations:** Department of Psychology, Brunel UniversityLondon, UK

**Keywords:** collectivism, gender role ideology, romantic beliefs, mate selection, marital difficulties

## Abstract

Previous studies have established that Indians tend to be greater in collectivism and gender role traditionalism than Americans. The purpose of the present study was to examine whether these differences explained further cultural differences in romantic beliefs, traditional mate preferences, and anticipation of future difficulties in marital life. Results revealed that Indians reported greater collectivism than Americans and, in turn, held stronger romantic beliefs. Additionally, Indians' greater collectivism and endorsement of more traditional gender roles in part predicted their preferences for a marital partner possessing traditional characteristics, and fully accounted for their heightened concerns about encountering future difficulties in marital life. These results shed light on the processes underlying cultural differences in relationship attitudes and preferences, and point to culture-specific therapies to enhance marital functioning.

## Introduction

Existing in almost all societies, the marital relationship is an important contributing factor to health and well-being (Williams et al., 1992). Through this union, new familial dynamics are configured and indelible bonds formed between individuals (Larson and Holman, 1994). Traditionally, marriages were characterized by clearly-defined gender roles: women assumed responsibility over domestic needs, while men were the primary breadwinners. Over the years, however, marital dynamics have shifted. Factors such as later onset of marriage, increased education, women's mounting independence, and higher demand for dual-earner households have redefined mate preferences and contributed to a growing need for changes in marital roles (Barnett and Hyde, 2001; Wierda-Boer et al., 2009).

Cultures vary widely in the norms, attitudes, and customs surrounding marriage and the roles of husbands and wives. In Eastern, collectivistic cultures, marriage is often viewed as a sacred institution (Marshall, 2008). Long-established norms and customs surround this practice, with sometimes strict cultural sanctions against those who defy these standards (Netting, 2010). While in Western, individualistic cultures it is generally left to the discretion of individuals to select their own marital partner, in Eastern, collectivistic cultures this process often involves the input of family members to ensure that the partner is a good fit within the family network (Myers et al., 2005).

Nevertheless, industrialization and globalization have increasingly blurred the lines between cultures around the world (Wang et al., 2010). More and more of today's young adults are redefining their beliefs about love and romance, their attitudes toward marital life, and what qualities they are seeking in a lifetime partner (Buss et al., 2001). The current study aimed to gain a deeper insight into these emerging changes and their influence on young adults' expectations for their future marital life. More specifically, we examined the influence of collectivism and gender role ideology on romantic relationships within two distinct cultural groups—Americans and Indians. India is considered one of the most collectivistic countries in the world (Hofstede, 1980; Buss et al., 1990), and Indians tend to endorse traditional gender roles (Suppal et al., 1996). In contrast, the United States is highly individualistic (Imada, 2012) and espouses flexible gender roles that are largely malleable to each couples' needs (Bartley et al., 2005). We assessed whether collectivism and/or gender role ideology explained potential differences in American and Indian participants' romantic beliefs, mate preferences, and anticipated future difficulties in marital life.

### Cultural dimensions: individualism, collectivism, and gender role ideology

People's attitudes and behaviors are shaped and directed by the norms and customs prevalent in their particular social milieu (Cialdini and Goldstein, 2004). Cultural values—in particular, individualism, and collectivism—influence how people define themselves, relate to others, and interact with their social environment (Triandis, 1995). Western, individualistic cultures emphasize the rights of the individual, advocating freedom of personal choice (Kashima et al., 1995; Buunk et al., 2008). This value system encourages independence, self-expression, and uniqueness. Individualists set meaningful personal goals, look within themselves to make decisions, and are guided by their own self-determination and life choices (Hagger et al., 2014). Personal needs frequently take precedence over group needs; social interactions are cultivated on the basis of one's own beliefs and motives, rather than maintained out of a sense of duty or social courtesy (Greenfield, 2013).

In contrast, many Eastern cultures stress the merits of in-group harmony and cohesion (Buunk et al., 2010; Imada and Yussen, 2012). The interdependent self, rather than regarded as a separate entity, is contextualized and defined by group membership (Markus and Kitayama, 1991). Social behavior is governed by the standards, customs, and duties set by the in-group (Lykes and Kemmelmeier, 2014). Therefore, conducting oneself in accordance with conventional customs is heavily stressed, and individuals risk criticism by community members if they stray too far from these expectations. Similarly, to retain group uniformity and preserve its structural integrity, collectivists tend to respect social order and the authority of elders, even at a cost to one's own choices (Nath and Craig, 1999). In as much as collectivists are socialized to consider the well-being of the group over their own needs, they are likely to abandon personal desires that conflict with group welfare (Le and Impett, 2013).

In addition to individualism and collectivism, cultural differences in gender role ideology may also influence close relationship processes. Gender role ideology refers to socially constructed beliefs about men and women's ideal characteristics, responsibilities, and conduct (Claffey and Mickelson, 2009). An egalitarian gender role ideology emphasizes similarity between the sexes, whereas a traditional gender role ideology emphasizes differences (Stanik and Bryant, 2012). Aggregating the personal beliefs held by individuals, cultures also vary in gender role ideology, influencing the way communities view men and women (Perrone-McGovern et al., 2014). These cultural norms can play a key role in marital relationships, shaping the ways that spouses behave toward each other, perceive the quality of their relationship, and divide up family responsibilities (Perry-Jenkins and Crouter, 1990).

### Predictors of romantic beliefs

Studies have shown that gender role ideology and collectivism may exert separate influences on relationship processes, such that gender role traditionalism strengthens romantic beliefs (Peplau et al., 1993), whereas collectivism weakens them (Medora et al., 2002). Romantic love, also referred to as passionate love, is thought to be a cultural universal (Hatfield and Rapson, 1987). Across cultures, there tends to be more similarities than differences in passionate love (Neto et al., 2000), suggesting that passion may have evolved across cultures to facilitate pair-bonding (Fisher, 2012). For example, Jankowiak and Fischer (1992) reported the occurrence of romantic love within 89% of their culturally-diverse participant sample. Moreover, some researchers have suggested that attraction and love develop between partners in a complementary manner: people seek partners possessing qualities that they themselves lack, thereby enhancing their sense of wholeness and well-being (Mathes and Moore, 1985; Richerson and Boyd, 2005; Eagly et al., 2009).

Similarly, traditional gender roles also emphasize complementarity; men and women are ascribed distinctive, but interdependent responsibilities based on their perceived aptitudes (Wolkomir, 2009). The yin and yang of male and female stereotypes—with men as dominant protectors, and women as sensitive and maternal—meant that heterosexual unions were thought to create an ideal romantic fit. For example, fairy tales, movies, and music often perpetuate the romantic belief that a knight in shining armor should rescue the damsel in distress. Peplau et al. (1993), in their 15-year study of dating, love, and marriage, found that women who endorsed a more traditional gender role ideology also reported stronger romantic love toward their partner. They further reported greater respect and admiration for their partners—qualities often associated with beliefs in romantic love.

In as much as traditional gender roles are more readily endorsed in collectivistic cultures (Sastry, 1999), one might expect that romantic ideals would also be stronger in this cultural milieu. However, many collectivistic cultures do not encourage romantic beliefs as a basis for marital partner selection (Levine et al., 1995). Therefore, romantic beliefs—a personal ideal—are viewed separately from the act of marriage—a social duty. In fact, these beliefs may be discouraged by elders if they threaten to interfere with familial or cultural duties when selecting a partner in line with social standards (Medora et al., 2002).

From an early age, children in collectivist cultures internalize the values of upholding family honor, following tradition, and showing respect to parents (Beilmann et al., 2014). As adolescents get older and the prospect of marriage looms larger, they are encouraged to put aside their personal desire for romance and intimacy, and embrace a more practical approach to relationships (Madathil and Benshoff, 2008). Given these conflicting ideals, we predicted that Indians would report greater gender role traditionalism than Americans, driving their romantic beliefs up, while their greater collectivism would simultaneously drive it down, creating opposing pressures on their endorsement of romantic beliefs.

### Predictors of marital mate preferences

People from Western, individualistic backgrounds tend to view a romantic relationship as an exclusive bond, formed between two individuals who share attraction and love, and serving their own personal needs (Moore and Leung, 2001; MacDonald and Jessica, 2006). As such, the qualities seen as desirable in a partner are a personal matter, arising from one's subjective preferences and ideals. In adolescence, Westerners usually begin exploring different romantic relationships through dating. This progression is not only customary, but commonly encouraged by the parents and family members of the young adult (Morgan et al., 2010). Therefore, Westerners are expected to initiate the process of mate selection themselves, ensuring compatibility and shared interests with their partner.

In contrast, marriage within Eastern, collectivistic cultures helps to reinforce family obligations as young adults are expected to marry in order to fulfill cultural and familial commitments (Zhang and Kline, 2009). Families are often included in the mate selection process from the very beginning (MacDonald et al., 2012). Parents and children filter through prospective marital candidates and find an appropriate partner who will provide a good fit with the family (Batabyal, 2001). Notably, a high degree of parental involvement in mate selection is still prevalent in India (Netting, 2010). Parents encourage children to adopt a pragmatic approach to marriage, giving weight to those qualities that are compatible with cultural and familial standards (Levine et al., 1995).

The strong emphasis on family values and conforming to the traditional conceptualization of marriage means that conventional gender roles tend to be endorsed in Eastern, collectivistic cultures (Sastry, 1999). In India notably, the rate of arranged marriage is especially high compared to other collectivistic nations. In this cultural milieu over 90% of marriages are organized by parents and elder family members (Uberoi, 2006). Therefore, culturally sanctioned objective criteria play an especially instrumental part in choosing suitable marital partners for young adults. Potential partners are scrutinized in terms of the various roles they will be fulfilling within the marriage: women are largely expected to carry out household and childrearing tasks, whereas men are expected to focus on meeting the financial necessities of the family (Suppal et al., 1996). Ultimately, partners are chosen and marital alliances are established on the presumption that each side will fulfill their respective obligations, thereby upholding the marriage and ensuring the smooth running of family life. Lalonde et al. (2004) found that second-generation South Asians living in Canada who were more interdependent desired more gender-traditional mate characteristics, such as a partner who would be a good provider or who possessed childrearing skills. In the same respect, we predicted that Indians would report greater gender role traditionalism and collectivism than Americans and, in turn, show stronger preferences for mate characteristics that are consistent with traditional gender roles.

### Predictors of anticipated marital difficulties

A large part of selecting the right marital partner and sustaining long-term relationship satisfaction is ensuring that both individuals can successfully negotiate the roles each one will play in the household (Hallett and Gilbert, 1997). In the Western world, women are more educated and career-oriented than in any other generation; they have a strong desire to expand beyond their traditional role as a homemaker (Barnett and Hyde, 2001). Meanwhile, men in Western cultures have progressively assumed responsibility for various domestic tasks that were traditionally undertaken by women. By performing gender-atypical chores, men are challenging their traditional gender roles and spurring the development of more egalitarian ideals (Pitt and Borland, 2008). According to Bianchi et al. (2005), the responsibilities of a couple have shifted immensely since the 1960s, with women cutting the time they spend on household chores by half, while men have nearly doubled their time.

Despite these changes in the Western world, Indian society has retained clear guidelines about the roles that men and women should play in the family (Andrade et al., 1999). Household chores are expected to be the wife's duty, while earning a living is considered the husband's role. Childrearing and family decision-making power also follows a traditional arrangement: whereas females are glorified for motherhood and take charge of children's day-to-day activities, fathers are considered the head of the household and take primary responsibility for decision-making on behalf of all family members (Sastry, 1999). These expectations often restrict personal choice and suppress individual ambitions within marital relationships. Instead men and women are pressured to conform to the gender division of labor, curbing their behavior to fit along these gendered lines—irrespective of personal desire—thereby permitting society to continue to regulate individual freedom and justify the separation between the sexes (Chafetz, 1990).

Collectivistic family values also encourage deference to older family members, with young couples often living with in-laws in extended family living arrangements (Georgas et al., 2001). While this may be a beneficial in certain respects, allowing for more help with daily activities and chores, it can put additional pressure on young couples to act in accordance with collectivistic cultural standards and customs (Singh, 2008). In India, for example, younger married couples who express a need for closeness and intimacy within the marital relationship are often met with disapproval and resistance by the in-laws they live with (Sandhya, 2009). This conflict, likewise, may evoke disagreement between the couple—especially in cases where one partner takes the in-laws' side (MacDonald et al., 2012). Older family members may feel that the couple's desire to make their own decisions undermines the long-standing authority of elders and disrupts the hierarchy of the household family system (Nath and Craig, 1999). Sonpar (2005), for example, found that many Indian couples who sought marital therapy were struggling to reconcile their collectivistic value of deference to parents and in-laws with their personal desire to strengthen their marital relationship.

For collectivist newlyweds, the initial stages of married life can be especially challenging for both the husband and wife as they try to adjust to their new roles within the family organization. Often, the new daughter-in-law's place in the familial hierarchy is at the very bottom of the system (Derne, 1994). Especially important is her new role as a dutiful and devoted wife. In India this connection of a wife to her husband is regarded as particularly important and expected to endure eternally (Sonpar, 2005). For instance, historically if a husband died before his wife it was not uncommon for a woman to practice Sati—a religious custom where a wife, in a show of ultimate devotion to her husband, sacrificed herself during her husband's funeral (Harlan and Courtright, 1995). Additionally, though, a newly married woman not only has to adjust to her role as a faithful wife, but she also has to take on the role of an obedient daughter-in-law to her new in-laws (Das, 1979). The demands of her new role can be difficult as she becomes primarily responsible for all the chores in the house and the general upkeep of the home. Having to be accountable to her mother-in-law can also be emotionally stressful with little defense from her husband. As one older Indian husband explained in clinical therapy, he felt helpless to step in and offer protection to his wife from the mistreatment she received at the hands of his mother in the early stages of their marital life because it was inconceivable to challenge the authority of parents (Sonpar, 2005).

In addition to these trials and tribulations, the new wife is also expected to taper off the relationship she enjoyed with her family of origin; her husband's household has become her primary family and her in-laws have replaced the parental figures in her life (Das, 1979). The husband, as well, goes through many adjustments; he needs to balance his initial relationship with his parents as a son with his new role as a husband who is starting a family of his own. While he may want to build a close relationship with his wife, he has to be careful not to become too devoted to her and risk hurting his image as a man who is in charge of his family and does not become easily persuaded by his wife's requests (Derne, 1994; Sonpar, 2005).

While Western, individualistic couples have gradually moved away from strictly-defined gender roles, negotiating among themselves what arrangements fit them best, couples from collectivist cultures may still struggle to adjust to established traditional customs, putting aside their own personal needs or desires for their marriage. In India, for example, rigid cultural rules continue to stress conformity to traditional gender roles (Das, 2011), leaving couples with very little room to deviate from conventional patterns as they try to adapt to marital life. Based on this rationale, we predicted that Indians, due to their stronger endorsement of traditional gender roles and collectivism, would anticipate facing more difficulties in their future marital life than Americans.

### The present research

A considerable body of research has been devoted to understanding cultural disparities in romantic relationships and family structuring (Lalonde et al., 2004; Buunk et al., 2010; Goodwin et al., 2012). While informative, this research has focused on married couples or university students, offering a glimpse into a specific sub-group of people within the wider cultural context. Although the spread of globalization has meant that the younger generation in Eastern, collectivistic societies are increasingly adopting Western notions of love, romance, and family structuring (Marshall, 2010), research based on this participant sample remains sparse. To better gauge these issues, the present study recruited unmarried young adults within two nations that strongly reflect individualistic vs. collectivistic ideals and egalitarian vs. traditional gender role ideologies—America and India, respectively.

*Hypothesis 1:* Compared to Americans, Indians' greater gender role traditionalism will drive up their romantic beliefs, while their greater collectivism will simultaneously drive them back down.

*Hypothesis 2:* Compared to Americans, Indians' greater gender role traditionalism and collectivism will mediate their greater preference for a marital partner with traditional characteristics.

*Hypothesis 3:* Compared to Americans, Indians' greater gender role traditionalism and collectivism will mediate their greater anticipation of future difficulties in marital life.

## Methods

### Ethics statement

The Brunel University Psychology Research Ethics Committee provided ethical approval for this study. Participants gave written informed consent at the beginning of the survey. All responses were confidential and kept anonymous.

### Participants

Two hundred and nine participants were recruited for this study (90 women and 119 men; mean age = 25.04, *SD* = 6.53) through Amazon's Mechanical Turk. They were paid $0.35 (USD) for completion of the survey. All participants were single; 82% indicated they wanted to get married in the future, while 14% were undecided, and the remaining 4% stated they were not interested in getting married. 69% of participants desired children in the future, 19% were undecided, and 12% did not want children. 51% of participants resided in India; of these participants, only one was not born in India, but had lived there for 20 years. The ethnicity of Indian participants consisted of 1% White/Caucasian and 99% South Asian. 49% of participants lived in the United States; of these participants, only three had been born outside of the US, but had lived there for an average of 28.33 years. American participants' ethnicity consisted of 81% White/Caucasian, 1% South Asian, 5% East Asian, 7% African/Caribbean, 3% mixed race, and 3% “other.”

### Procedure and materials

An online survey was generated through a survey-development website (www.surveymonkey.com). Participants first completed demographic questions before moving on to the following measures.

#### Gender role ideology

The 20-item Attitudes Toward Sex Roles Scale (Larsen and Long, 1988) assessed the endorsement of a traditional gender role ideology. Example items include, “In groups that have male and female members, it is more appropriate that leadership positions be held by males” and “Almost any woman is better off in her home than in a job or profession.” Participants used a 5-point Likert scale (1 = *Strongly disagree*, 5 = *Strongly agree*) to indicate their level of agreement with each item. Cronbach's alpha was 0.93 for Americans and 0.64 for Indians.

#### Collectivism

Eight items from the Horizontal/Vertical Individualism/Collectivism Scale (Sivadas et al., 2008) measured collectivism in two domains: cooperation and dutifulness. An example item is “I would do what would please my family, even if I detested that activity.” Responses were measured on a 5-point Likert scale ranging from 1 *(Strongly disagree)* to 5 *(Strongly agree)*. We collapsed across the horizontal-vertical dimension to increase the reliability of the collectivism scale (*a* = 0.79 for Americans and *a* = 0.82 for Indians).

#### Romantic beliefs

The 15-item Romantic Beliefs Scale (Sprecher and Metts, 1989) consists of four subscales: *Love Finds a Way*, One *and Only*, *Idealization* and *Love at First Sight*, each measured on a 7-point Likert scale (1 = *Strongly disagree*, 7 = *Strongly agree*). A sample item from the *Love Finds a Way* subscale is, “If I love someone, I know I can make the relationship work, despite any obstacles.” Internal consistency for three of the subscales was good, ranging from 0.83 to 0.86; however, for the subscale *Love at First Sight*—consisting of three items—internal consistency was only 0.31 for Americans and 0.12 for Indians. Given the low reliability of the *Love at First Sight*, we excluded this subscale and calculated the total score for the remaining three subscales, utilizing this total score in the analyses. The internal consistency for this total score was *a* = 0.90 for Americans and *a* = 0.89 for Indians.

#### Mate preferences

The 27-item Essential Characteristics of a Spouse Scale (Gilbert et al., 1991) measured the extent to which a range of mate characteristics are desirable in a future spouse. Example items include, “Someone who enjoys the same recreational activities,” and “Someone who makes me feel protected and secure.” Four additional items were added because of their potential relevance for choosing a mate in traditional, collectivistic societies. In accordance with measures of mate preferences by Buss et al. (1990) and Lalonde et al. (2004), these items were, “Comes from a family with a good reputation,” “Favorable social status or rating,” “Similar religious background,” and “Someone my family approves of.” Participants used a 5-point Likert scale (1 = *Not at all important*, 5 = *Essential*) to indicate their level of agreement with each item. Principal components analysis with oblique rotation produced a two-factor solution. The first factor, accounting for 29.4% of the variance, reflected non-traditional mate characteristics (20 items), while the second factor, accounting for 15.5% of the variance, reflected traditional mate characteristics (10 items). All items loaded at 0.35 or higher on their respective factor. Cronbach's alpha was 0.91 for Americans and Indians respectively in non-traditional mate characteristics and 0.88 for Americans and 0.84 for Indians in traditional mate characteristics. Given that none of the independent variables were significant predictors of non-traditional mate characteristics, we do not discuss this variable further.

#### Anticipated future difficulties in marriage

Consisting of 16 items, the Future Difficulties Scale (Gilbert et al., 1991) measured the issues participants anticipated facing in their future marital life. The measure consists of three subscales: *Childcare*, *Sharing Family Work*, and *Career Advancement*. Instructions asked participants to reflect on each item and indicate how likely a barrier or difficulty such a situation might pose in their future marital life. Ratings were made on a 5-point Likert scale (1 = *Unlikely a difficulty for me, 5 = Very likely a difficulty for me)*. Example items include, “Having to work more than I want to for financial reasons” and “Pursuing a career compatible with my interests and abilities despite family demands (financial or otherwise).” Although the internal consistency for each of the subscales was reasonable, ranging from 0.71 to 0.83, the overall scale was more reliable (*a* = 0.92 for Americans and *a* = 0.87 for Indians). Therefore, the total score for anticipated future difficulties was utilized in the analysis.

## Results

Means and standard deviations are reported in Table 1, and Pearson's correlations are reported in Table 2. We created an effect coded variable to distinguish between our two cultural groups (1 = Indian, −1 = American). Age and sex (1 = male, −1 = female) were also controlled in the following models.

**Table 1:** **Descriptive statistics**.

	**Americans**		**Indians**		**t_(209)_**
	***M***	***SD***	***M***	***SD***	
Age	25.60	8.02	24.70	4.68	1.01^**^
Gender role ideology	40.79	16.05	59.94	7.87	−10.74^**^
Collectivism	26.57	5.42	30.22	4.98	−5.10
Romantic beliefs	56.78	13.82	63.77	10.81	−4.02^*^
Traditional mate characteristics	28.09	9.16	38.12	6.63	−9.02^**^
Future difficulties	37.55	11.08	44.13	8.40	−4.75^*^

^†^p < 0.10;

* p < 0.05;

** *p < 0.01*.

**Table 2:** **Pearson's correlations for Indians and Americans**.

**Variable**	**1**	**2**	**3**	**4**	**5**	**6**	**7**
Sex		0.02	0.26^**^	−0.04	−0.15	−0.04	0.12
Age	0.04		0.03	−0.02	0.11	0.09	0.19^†^
Gender role ideology	0.42^**^	0.06		0.02	−0.09	0.32^**^	0.48^**^
Collectivism	0.14	−0.06	0.37^**^		0.61^**^	0.48^**^	0.31^**^
Romantic beliefs	0.02	−0.26^**^	0.15	0.33^*^		0.18^†^	0.06
Traditional mate characteristics	0.19^†^	−0.04	0.60^**^	0.45^**^	0.24^*^		0.50^**^
Future difficulties	0.03	−0.08	0.33^**^	0.34^**^	0.07	0.43^**^	

*Americans' data are presented below the diagonal, and Indians' data are presented above the diagonal*.

† p < 0.10;

* p < 0.05;

** *p < 0.01*.

To test the relationship between culture and our respective dependent variables, three analyses were conducted via a bootstrap method for testing multiple mediation effects (Preacher and Hayes, 2008). A mediational model tests the association between an independent variable and a dependent variable through a third variable, known as the mediator or the suppressor. To establish a mediational (or indirect) effect, the association between the independent and dependent variable—the *total effect*—must be larger than the association between the independent and dependent variable after controlling for the mediator—the *direct effect* (MacKinnon et al., 2000). In these analyses, culture was the independent variable, collectivism, and gender role ideology were the mediators, and romantic beliefs, traditional mate preferences, and anticipated future difficulties in marital life were the dependent variables.

**Figure 1:**
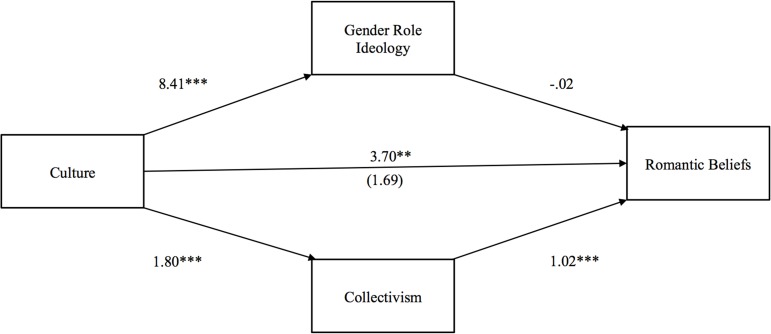
**Indirect effect of culture on romantic beliefs through gender role ideology and collectivism**. The value in parentheses represents the direct effect, and the value directly above is the total effect. ^*^p < 0.05, ^**^p < 0.01, ^***^p < 0.001.

The first model tested Hypothesis 1—that Indians would report greater gender role traditionalism than Americans, thereby driving their romantic beliefs up, while their greater collectivism would simultaneously drive these beliefs down. As seen in Figure 1, the total effect of culture on romantic beliefs (i.e., not controlling for collectivism or gender role ideology) was larger and significant (*b* = 3.70, *p* < 0.001) compared to the direct effect (*b* = 1.69, *p* > 0.05)^1^. Examination of the 95% bias-corrected confidence intervals (CI) from 5000 bootstrap samples revealed that the indirect effect of culture on romantic beliefs through gender role ideology was not significant [*b* = 0.13 (*CI*: −0.99, 1.44)]. On the other hand, the indirect effect through collectivism was significant [*b* = 1.84 (*CI*: 0.96, 3.06)], partially confirming our hypothesis, although not in the direction originally predicted.

**Figure 2:**
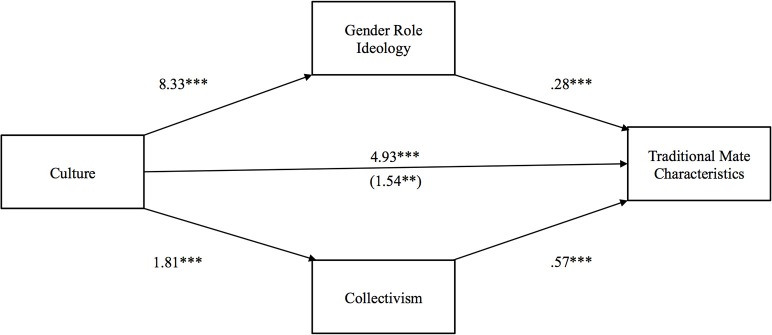
**Indirect effect of culture on preferences for traditional mate characteristics through gender role ideology and collectivism**. The value in parentheses represents the direct effect, and the value directly above is the total effect. ^*^p < 0.05, ^**^p < 0.01, ^***^p < 0.001.

The second hypothesis tested whether Indians' more traditional gender role ideology and collectivism would explain why they preferred more traditional mate characteristics than Americans. Partly confirming our hypothesis and demonstrating partial mediation, as reported in Figure 2, the total effect of culture on preferences for traditional mate characteristics (*b* = 4.93, *p* < 0.001) was larger than the direct effect (*b* = 1.54, *p* < 0.01). The indirect effect of culture on traditional mate characteristics through traditional gender role ideology was significant [*b* = 2.34 (*CI*: 1.60, 3.33)], as was the indirect effect of collectivism [*b* = 1.01 (*CI*: 0.60, 1.60)], showing that both mediators exerted separate, positive influences on the relationship between culture and preferences for traditional mate characteristics.

**Figure 3:**
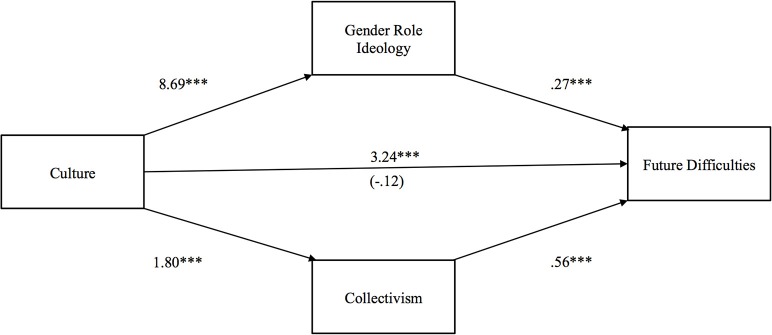
**Indirect effect of culture on future difficulties on marital life through gender role ideology and collectivism**. The value in parentheses represents the direct effect, and the value directly above is the total effect. ^*^p < 0.05, ^**^p < 0.01, ^***^p < 0.001.

Our third hypothesis proposed that Indians, due to their more traditional gender role ideology and greater collectivism, would anticipate facing more difficulties in their future marital life than Americans. Figure 3 shows that the total effect of culture on future difficulties was positive and significant (*b* = 3.24, *p* < 0.001), whereas the direct effect was negative and not significant (*b* = −0.12, *p* > 0.10). Fully corroborating our hypothesis, the indirect effects of culture on anticipated future difficulties through gender role ideology [*b* = 2.32 (*CI*: 1.40, 3.52)] and collectivism [*b* = 0.95 (*CI*: 0.52, 1.75)] were both positive and significant.

## Discussion

The purpose of this research was to test whether gender role ideology and collectivism mediated the associations of culture with romantic beliefs, mate preferences, and future difficulties in marital life. On the whole, we found that Indians reported greater collectivism and, in turn, more romantic beliefs, more traditional mate preferences, and greater anticipation of future difficulties in marriage. Indians also endorsed a more traditional gender role ideology compared to the American group, which explained their stronger preferences for traditional mate characteristics and greater anticipation of future difficulties in their marital life. The main findings of this study add to the current literature on mate selection and marital relationships by providing evidence that both gender role ideology and collectivism exert unique influences on relationship attitudes and preferences. In the following sections, we discuss these findings in greater detail.

Historically, it was suggested that a successful marriage is rooted in the complementary nature of male and female qualities. For example, Fromm (1956) argued that the frisson generated by masculine and feminine qualities enhanced romantic love and emotional fulfillment in heterosexual relationships. From ancient Sanskrit texts to the love songs of medieval troubadours, from Hollywood to Bollywood, traditional romantic beliefs reflect the trope of the chivalrous male and the receptive female. More recently, however, roles among the sexes have shifted, conceivably redefining the ideals that are sought in a mate. Indeed, Buss et al. (2001) found a notable shift in mate preferences over a 57-year time period. Gender-related traits—such as cooking skills, housekeeping abilities, and chastity—became less important, and men and women increasingly converged in their preferences over time. Buss et al. (2001) also found that mutual attraction became increasingly important to both sexes by the end of the 20th century.

To expand upon this area of research, we examined gender role ideology as a mediator of the association between culture and romantic beliefs. No significant results were found. In past generations, distinct gender roles were strongly emphasized, and men and women were more likely to be venerated for how well they could fill their respective roles (Cherlin, 2005). However, in today's society, notions of masculinity, and femininity have shifted, allowing for men and women to abide by less-defined gender roles. Therefore, the ideals of what make a successful union and generates love between two individuals may have also deviated from what was assumed important in past generations.

We also examined collectivism as a mediator of the association between culture and romantic beliefs. In contrast to Western cultures, marriage is often regarded as a necessary practice in Eastern, collectivistic cultures, and young adults are expected to marry as part of their duty to culture and family (Netting, 2010). Consequently, with the involvement of other family members and the purpose of marriage heavily rooted in family obligations, intimacy between partners is not a necessary requirement of marital bonds (Myers et al., 2005). Nevertheless, while love may not be the primary selection criterion for a marital partner, this does not necessarily dispel the desire for it. Indeed, in many Indian classic folklores and modern Bollywood movies, romantic love is often held in high esteem (Chakraborty, 2010). In these epics, couples who are brought together through romantic love are frequently revered. However, these same stories also warn of the perils of romantic love and its potential to be destructive toward family ties (Gala and Kapadia, 2014). Thus, while collectivistic values may emphasize more practicality in marital partner selection and relationship maintenance, actual idealistic views on love and romance may be strongly supported within the cultural milieu. In line with this reasoning, Neto (2007), in his cross-cultural study of love styles, concluded that Indians were higher in pragmatic love, but they also did not differ from Americans or Portuguese in Eros (i.e., passionate love). In a recent study, Gala and Kapadia (2014) found that while commitment to a relationship is very important to emerging adults in Indian, so is love. Indian participants expressed strong support for romantic love and believed it should be an integral part of married life; even in cases of arranged marriage, participants trusted that love would develop between partners over time.

Contrary to our predictions, but consistent with these studies, we found that Indians' stronger romantic beliefs were driven by their greater collectivism. While the purpose of marriage may differ within collectivistic and individualistic cultures, leading collectivists to be more pragmatic in their search for a marital partner (Levine et al., 1995), the actual desire for love may not differ so much from Westerners. Moreover, the cultural sanctions against the expression of romantic love may actually enhance the desire for it relative to Westerners, who may freely express it without fear of reproach by the community. Note, however, that, our study measured the romantic beliefs of unmarried participants, not their actual experience of love within a marital relationship. It may be that the romantic ideals of unmarried Indians may not be realized within an actual marriage, especially if it is arranged. Future studies assessing romantic ideals pre- and post-marriage could help shed further light on this area of study.

We further examined the predictors of spousal preference. Our findings showed that Indians reported a more traditional gender role ideology and greater collectivism; in turn, they showed a stronger preference for traditional mate characteristics in a marital partner.

As mentioned previously, ideals in mate preferences and what is thought to be necessary for relationship longevity and satisfaction may have shifted over the years (Hatfield and Rapson, 2006). For instance, in contemporary India, children more than ever are expressing their desire for a partner who is compatible with them on an individual level. Therefore, among the traditional concerns of selecting someone who is socially suitable, they are also increasingly seeking a mate who can meet their personal needs for connection and intimacy. Shukla and Kapadia (2007) recount the comments of one of their participants, who expressed an interest in finding a girl with a good nature and disposition. If she possessed these characteristics, he explained, he was not too concerned about her family background.

Nevertheless, while Westernization may be inspiring young adults to take a stronger stance when choosing a personally-compatible partner, this has not necessarily reduced the importance still afforded to traditional criteria. For instance, the caste system, particular to India, is still commonly applied when choosing marital partners (Dhar, 2013). Banerjee et al. (2013) found that within the matrimonial advertisements that are being increasingly used for finding a marital partner, ads are organized under caste headings, allowing those who are seeking a marital mate to, first and foremost, locate someone within their own caste. Providing dowry is also reminiscent of traditional marital considerations when assessing suitable matches. While this practice is no longer considered legal in India today, it is still largely practiced out of social courtesy (Sonawat, 2001). Thus, while individually-calibrated factors such as personality and charisma are now being incorporated into the partner selection process, cultural, and familial input still continues to be very important.

Furthermore, Hinduism endorses a patriarchal belief system and the preservation of family lineage (Netting, 2010). Premarital sex is prohibited and marriage is viewed as the framework for upholding the family structure; accordingly, casual dating is largely considered taboo (Manohar, 2008). Without the opportunity to initiate a personal connection, prospective mates are instead evaluated in terms of multiple pragmatic qualities, such as one's economic, social, and religious background. In line with our results, Buss et al. (1990) also found that Indian participants showed a stronger preference for traditional values such as chastity and the desire for home and children.

Consistent with other research, we found that Indians' collectivism and gender role traditionalism contributed to their greater concerns about future difficulties in their marital life (Suppal et al., 1996; Sastry, 1999). Collectivistic cultural values of family honor and deference to older family members places additional pressure on married couples to maintain traditional gender roles, while economic needs and social advancements may necessitate otherwise. For example, Krishnan et al. (2010) found that despite Indian women's more readily-available job opportunities, they often felt ambivalent about working given the challenge it posed to conventional power dynamics. In fact, 47% of wives stated that they did not work because their husbands would not allow them.

Community pressures may also reinforce traditional conceptualizations of marriages, adding to the difficulty of adjusting to marital life for many Indian couples. George (2006) reported that working class Indian men who did not earn a sufficient income—obliging their wives to work menial jobs and become the household breadwinner—were viewed as “weak” and held in contempt by community members. These men were seen as breaking cultural norms that emphasize the male provider role, thereby bringing shame onto themselves and their families. In a society where reputation in the community and good family relations are vital, couples who deviate from acceptable role patterns run the risk of alienating family members, losing critical support and being ridiculed in the community. These fears inadvertently place pressure on Indian couples to maintain traditional marital dynamics irrespective of their personal desires.

## Limitations and future directions

Although our findings offer important insights into cultural influences on romantic beliefs, partner selection, and anticipated future difficulties in marital life, there are several limitations to this study that warrant discussion. While some studies, including our own, have shown that Indian participants express similar or even stronger romantic ideals or passionate love compared to their Western counterparts (Schmitt et al., 2004; Neto, 2007), other research has found otherwise (Medora et al., 2002). For example, irrespective of cultural background, Regan et al. (2004) found that 85% of their adolescent participants had experienced romantic love. On the other hand, Twamley (2013) found that Indians were suspicious and disapproving of premarital love that was thought to arise from “love at first sight” and included physical intimacy; however, “pure love” that abided by cultural and familial standards was deemed important and desirable in a relationship, especially within a marital context. Therefore, to clarify the mixed findings on this topic, it would be helpful for future research to take age, marital status, and love styles into account when exploring cultural differences in romantic beliefs and passionate love. Likewise, our research measured romantic beliefs rather than participants' actual experience of romantic love within relationships—a distinction that would be beneficial to consider in upcoming studies.

An additional limitation of this study was that we asked participants to rate their perceptions of how difficult they thought their future marital life would be. Further research should examine the *actual* difficulties experienced by married couples. Moreover, our research only focused on single participants. Research has shown that spouses who discussed their respective viewpoints on how to manage household labor, career goals, and parenting issues prior to getting married expressed greater satisfaction in their marriage (Hallett and Gilbert, 1997). It might also be that instrumental family support, especially for the collectivistic Indian sample, could help offset some of these difficulties. However, without taking these additional variables into account, our participants could only report what they foresaw their future difficulties might be instead of their actual experiences and challenges in marital life.

The researchers also noted the possibility that Indians may have anticipated greater future marital difficulties because they may be less prone to a positivity bias than Americans. However, while Indian participants showed a negative evaluation toward future marital circumstances, they also showed a positive evaluation of romantic beliefs, demonstrating that they are not generally showing a negativity bias by swaying toward bleaker thought or evaluation patterns. Finally, it is important to acknowledge that our sample of Indian participants, given their ability to speak English and have access to computers, may have been more educated and come from higher socioeconomic backgrounds compared to the average Indian living in India. Therefore, the participants in our study may not necessarily represent an accurate reflection of the more general Indian population. Likewise, the average age of participants in this study was in their mid-twenties. One might expect age to be positively correlated with gender role traditionalism, such that older generations are more traditional than younger generations, however, neither gender role ideology nor collectivism was significantly associated with age in our study. Future research may wish to compare gender role ideology and collectivism among younger and older generations to ascertain the influence of societal shifts on romantic beliefs and relationship dynamics between varying age groups.

## Concluding remarks

The current research sought to “unpack” the influence of culture on romantic beliefs, mate preferences, and anticipated future difficulties in marital life by examining the mediating roles of collectivism and gender role ideology. Contrary to past research that deemed romantic love as less important in collectivistic cultures, our findings suggested that today's generation of Indian youth actually possessed *stronger* romantic ideals than did their American counterparts. While it is still crucial for collectivist youth to be pragmatic in their mate choices, this does not detract from their desire for love and romance. We further found that Indians' gender role traditionalism and collectivism were associated with stronger desires for a partner with traditional mate characteristics and greater anticipation of future difficulties in marital life. Future research would profit from examining the ways that Indians' aspirations to abide by cultural customs and choose a marital partner according to family expectations can be reconciled with the demands of globalization, economic development, and political and social reforms in a changing society. These findings can aid in the development of culture-specific marital therapies that are based on the understanding and appreciation of different practices and norms across cultures.

### Conflict of interest statement

The authors declare that the research was conducted in the absence of any commercial or financial relationships that could be construed as a potential conflict of interest.

## Footnotes

^1^ When the independent variable is dichotomous, Preacher and Hayes (2008) recommend reporting unstandardized regression coefficients.
